# Cancer-associated fibroblast-related gene signatures predict survival and drug response in patients with colorectal cancer

**DOI:** 10.3389/fgene.2022.1054152

**Published:** 2022-11-25

**Authors:** Lei Zhang, Chao Xu, Si-Han Wang, Qin-Wen Ge, Xiao-Wei Wang, Pan Xiao, Qing-Hua Yao

**Affiliations:** ^1^ Department of Integrated Chinese and Western Medicine, The Cancer Hospital of the University of Chinese Academy of Sciences (Zhejiang Cancer Hospital), Institute of Basic Medicine and Cancer (IBMC), Chinese Academy of Sciences, Hangzhou, China; ^2^ The Second College of Clinical Medicine, Zhejiang Chinese Medical University, Hangzhou, China; ^3^ The First College of Clinical Medicine, Zhejiang Chinese Medical University, Hangzhou, China; ^4^ Department of Plastic Surgery, Zhejiang Hospital, Hangzhou, China; ^5^ Integrated Traditional Chinese and Western Medicine Oncology Laboratory, Key Laboratory of Traditional Chinese Medicine of Zhejiang Province, Hangzhou, China; ^6^ Key Laboratory of Head and Neck Cancer Translational Research of Zhejiang Province, Hangzhou, China

**Keywords:** colorectal cancer, cancer-associated fibroblasts, tumor microenvironment, prognosis, WGCNA

## Abstract

**Background:** Cancer-associated fibroblasts (CAFs) play an important role in the tumorigenesis, immunosuppression and metastasis of colorectal cancer (CRC), and can predict poor prognosis in patients with CRC. The present study aimed to construct a CAFs-related prognostic signature for CRC.

**Methods:** The clinical information and corresponding RNA data of CRC patients were downloaded from The Cancer Genome Atlas (TCGA) and Gene Expression Omnibus (GEO) databases. The Estimation of STromal and Immune cells in MAlignant Tumor tissues (ESTIMATES) and xCell methods were applied to evaluate the tumor microenvironment infiltration from bulk gene expression data. Weighted gene co-expression network analysis (WGCNA) was used to construct co-expression modules. The key module was identified by calculating the module-trait correlations. The univariate Cox regression and least absolute shrinkage operator (LASSO) analyses were combined to develop a CAFs-related signature for the prognostic model. Moreover, pRRophetic and Tumor Immune Dysfunction and Exclusion (TIDE) algorithms were utilized to predict chemosensitivity and immunotherapy response. Human Protein Atlas (HPA) databases were employed to evaluate the protein expressions.

**Results:** ESTIMATES and xCell analysis showed that high CAFs infiltration was associated with adverse prognoses. A twenty-gene CAFs-related prognostic signature (CAFPS) was established in the training cohort. Kaplan-Meier survival analyses reveled that CRC patients with higher CAFs risk scores were associated with poor prognosis in each cohort. Univariate and multivariate Cox regression analyses verified that CAFPS was as an independent prognostic factor in predicting overall survival, and a nomogram was built for clinical utility in predicting CRC prognosis. Patients with higher CAFs risk scores tended to not respond to immunotherapy, but were more sensitive to five conventional chemotherapeutic drugs.

**Conclusion:** In summary, the CAFPS could serve as a robust prognostic indicator in CRC patients, which might help to optimize risk stratification and provide a new insight into individual treatments for CRC.

## Introduction

Colorectal cancer (CRC) is globally the third most commonly diagnosed cancer and the second leading cause of cancer-related deaths (Bray et al., 2018). Although 39% of patients diagnosed with localized CRC present 90% 5-year survival, this decreases to 71% for patients with tumors that have spread regionally and is less than 14% in those with advanced distant metastases (Thompson et al., 2022). According to the Global Cancer Observatory (https://gco.iarc.fr/today), there were 555,628 new cases and 283,751 deaths from CRC in China in 2020. The occurrence and development of CRC is a multi-step and complex process with multiple genes involved. Colorectal cancer cells have an extraordinary biological ability to adapt themselves to adverse environments, leading to their strong invasive and metastatic characteristics (Kleppe et al., 2018). Conventional assessment, including methods based on tumor-node-metastasis (TNM) staging and pathology, is intrinsically subjective and not sufficient to predict treatment response and prognosis. The development of a novel prognostic model is therefore imperative for CRC. Prognostic prediction models are widely utilized both in the clinic and research to predict the probability or the risk of a specific events or future outcomes (Toll et al., 2008).

Cancer arises from the accumulation of gene mutations within cancer cells, while both tumorigenesis and patients’ response to therapies are strongly regulated by non-mutant cells and the extracellular matrix (ECM) within the tumor microenvironment (TME). Cancer-associated fibroblasts (CAFs) are a special type of fibroblasts that surround tumors and form a key part of the TME. In recent years, CAFs have received increasing attention due to their crucial roles in tumor invasion, angiogenesis, and ECM remodeling by promoting cell-cell interaction and the secretion of pro-invasive factors (Villaronga et al., 2018; Bertero et al., 2019). Targeting CAFs by altering their numbers, subtype or biological functionality is emerging as an attractive avenue to improve therapeutic strategies for cancer.

**FIGURE 1:**
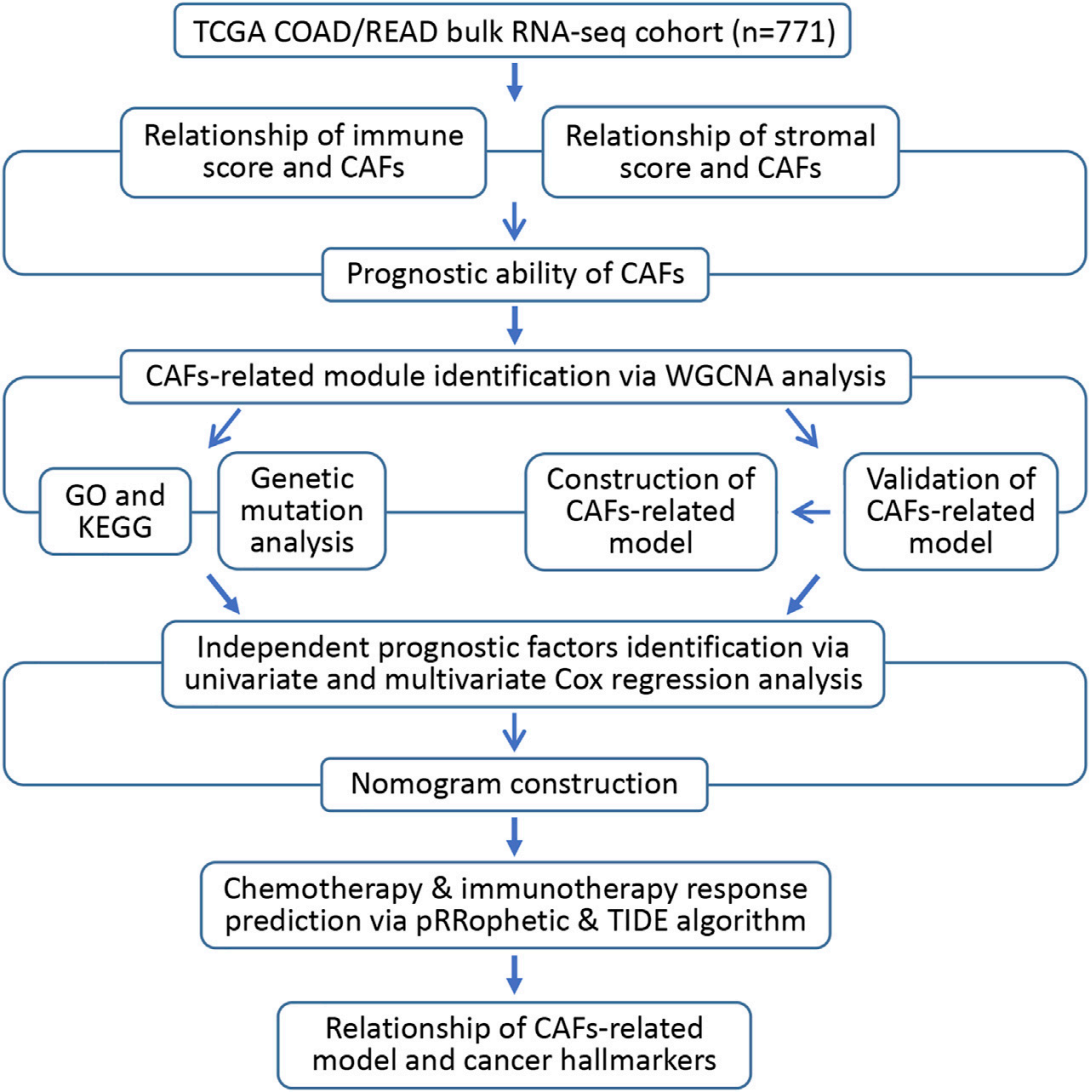
Flow diagram of the current investigation.

In this study, we identified the infiltration score of CAFs in CRC as a risk factor. The bulk transcriptome RNA-seq and relevant clinical data of CRC patients were obtained from The Cancer Genome Atlas (TCGA) datasets through the UCSC Xena browser (https://xenabrowser.net/datapages/). (Goldman et al., 2020) In addition, through a variety of bioinformatics methods, we aimed to discover promising CAFs-targeting therapeutic hallmarks and constructed a robust CAFs-related gene signature to predict the prognosis and drug response of CRC patients. Figure 1 illustrates the workflow of the study.

## Materials and methods

### Data source and preprocessing

Data that containing the RNA expression profiles and relevant clinical information of colon adenocarcinoma (COAD) and rectal adenocarcinoma (READ) patients were accessed through TCGA datasets. Following the removal of the batch effects, the two parts of data were merged using the “limma” R package (version 3.52.2). The GSE159216 and GSE72968 datasets were obtained from the Gene Expression Omnibus (GEO) data portal (https://www.ncbi.nlm.nih.gov/geo/) for further validation. In total, the data of 771 patients from the TCGA cohort, 283 patients from the GSE159216 cohort, and 585 patients from the GSE72968 cohort were recorded for utilization in the current study.

### Estimation of tumor microenvironment infiltration

The R package “xCell” (version 1.1.0) estimates the comprehensive levels of 64 immune and stromal cell types (Aran et al., 2017). The estimation of STromal and Immune cells in MAlignant Tumor tissues using the Expression data (ESTIMATES) algorithm (version 1.34.0) can accurately quantify the immune score and stromal score to identity the infiltration degree of immune cells and predict the immune status (Yoshihara et al., 2013). We applied xCell and ESTIMATES to separately calculate the abundance scores for stromal cells and immune cells for patient samples from different CRC stages.

### Construction of prognostic tumor immune cells

Spearman’s correlation analyses were conducted to determine the correlation between TME infiltration (immune score and stromal score) and CAFs levels in CRC samples. The “ggpubr” R package (version 0.4.0) was used to produce correlation plots. After selecting the *cut-off* values with the best sensitivity and specificity, CRC samples in the TCGA cohort were divided into two groups according to CAFs infiltration score using the “survival” R packages (version 3.6.1). The Kaplan-Meier survival curve was plotted using “survminer” R packages (version 0.4.6) to compare the survival rate.

### Identification of the hub CAFs-related module by weighted gene co-expression network analysis

In order to find genes that closely related to CAFs, we performed WGCNA by utilizing the “WGCNA” R package (version 1.69) to find modules highly correlated with CAFs levels and stromal score (Langfelder and Horvath, 2008). After calculating the Pearson correlation between each gene pair, the weighted adjacency matrix was constructed using the WGCNA function adjacency function. Then, we used topological overlap matrix analysis to cluster the adjacency matrix of CRC patients’ gene expression data. Next, the dynamic tree cut algorithm was applied to identify modules on the dendrogram. Finally, we calculated the correlation between the identified gene modules identified and CAFs levels to mine the hub module for subsequent analysis.

### Function and pathway enrichment analysis of genes in the hub module

In order to explore the biological function and pathway of genes in the hub module, the Gene Ontology (GO) and Kyoto Encyclopedia of Genes and Genomes (KEGG) pathway enrichment were analyzed and visualized through the “clusterProfiler” (version 3.18.0) and “org.Hs.eg.db” (version 3.1.0) R packages.

### CAFs-related genetic mutation analysis

The genetic landscape of CAF-related genes with copy number variations (CNV) and single nucleotide variations (SNV) from the TCGA datasets was generated with the “maftools” R package (version 2.6.05). Then, CNV and SNV correlation heatmaps were drawn using the “Complexheatmap” R package (version 2.6.2).

### Construction and validation of a CAFs-related prognostic model

We designed a prognostic signature for CRC patients by focusing on CAFs marker genes, which were identified from the hub module. Univariate Cox analysis of the overall survival (OS) was applied to screen the prognostic values of CAFs-related genes. Genes with *p* < 0.05 in the univariate Cox analysis were regarded as candidate prognostic genes. Next, we displayed the prognostic genes in a forest plot using the “forestplot” R package (version 1.9). To minimize the risk of overfitting, we used LASSO-penalized Cox regression analysis to eliminate genes with an overfitting tendency and built a prognostic signature using the “glmnet” R package (version 2.2.1) (Simon et al., 2011). The signature of CAFs was established as follows: CAFs risk score = Ʃ(β_i_*Exp_i_), where β_i_ represented the corresponding regression coefficients of each candidate prognostic gene, and Exp_i_ was the candidate gene’s expression value. According to the median value of CAFs risk scores, we divided CRC patients into high-risk and low-risk groups. The OS curve was plotted *via* Kaplan-Meier analysis. Meanwhile, time-dependent receiver-operating characteristic (ROC) analysis was carried out by the “survivalROC” R package (version 1.34.0). Finally, heatmaps were generated to visualize the association between the risk scores of CAFs and candidate genes. Similarly, we validated our CAF prognostic model on the GSE159216 and GSE72968 external validation cohorts.

### Construction of predictive nomogram

Univariate and multivariate Cox regression analyses were performed to identify the independent prognostic factors. A nomogram was then constructed based on CAFs signature, clinical stage, TNM stage and lymphatic invasion using the “rms” R package (version 6.0.1). Afterwards, the ROC curve and calibration curve were employed to evaluate the nomogram’s predictive performance and accuracy.

### Prediction of patients’ drug response based on CAFs signature

We predicted the chemosensitivity/resistance for the high and low risk groups *via* the “pRRophetic” R package (version 0.5). According to ridge regression, the half-maximal inhibitory concentrations (IC50) were estimated for TCGA samples (Geeleher et al., 2014a; Geeleher et al., 2014b). Furthermore, the Tumor Immune Dysfunction and Exclusion (TIDE) (http://tide.dfci.harvard.edu/) algorithm was employed to predict the potential response to immune checkpoint blockade (ICB) therapy between the two groups (Jiang et al., 2018).

### Association of prognostic CAFs signature with cancer hallmarks

A total of 50 hallmark gene sets were downloaded from the molecular signature database (MSigDB, http://software.broadinstitute.org/gsea/msigdb). These 50 hallmark gene sets were subjected to the gene set variation analysis (GSVA) R package (version 1.32.0) to further obtain the GSVA scores of each gene set for each sample from the TCGA cohort (Hänzelmann et al., 2013). The Pearson correlation between CAFs signature and 50 hallmark gene sets were calculated by the “Hmisc” R package (version 4.4.1).

### Human protein atlas database and immunohistochemistry verification

In order to further validate the protein expressions of CAFs signature genes, the immunohistochemistry staining images of prognosis-related genes in CRC tissues were retrieved from the HPA online database (http://www.proteinatlas.org/) (Uhlén et al., 2015).

### Statistical analysis

All statistical analyses were carried out using R software (version 4.0.3). The Wilcoxon signed rank test was applied for comparisons between two groups, and the Kruskal-Wallis test for comparisons between three or more groups. Statistical significance was determined as two-sided with *p* < 0.05.

## Results

### TME infiltration patterns with different CRC stages

**FIGURE 2:**
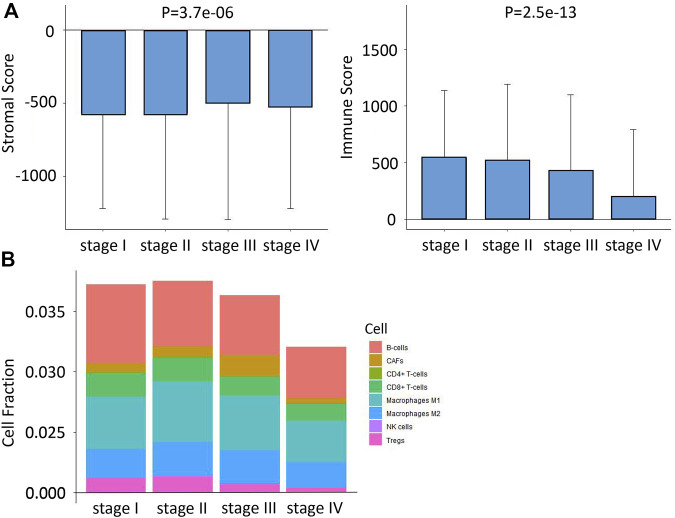
Differences in immune infiltration characteristics among the four stages. **(A)** Differences in stromal score and immune score among the four stages based on xCell algorithms. **(B)** Differences in eight immune cell scores among the four stages based on ESTIMATE algorithms.

By running xCell and ESTIMATES algorithms, we measured the TME constituents in patients with different stages of CRC from the TCGA cohort. As shown in Figure 2, the pooled results of the stacked bar graph and Wilcoxon analyses on TCGA COAD/READ datasets revealed the stromal and immune scores; the infiltrations of several TME contents such as B cells, CD8^+^ T cells, M1 Macrophages, M2 Macrophages, NK cells and Tregs were lower at later stages of CRC. However, the infiltration of CAFs increased first and then decreased in stage IV.

### Clinical correlation of tumor-infiltrating CAFs

**FIGURE 3:**
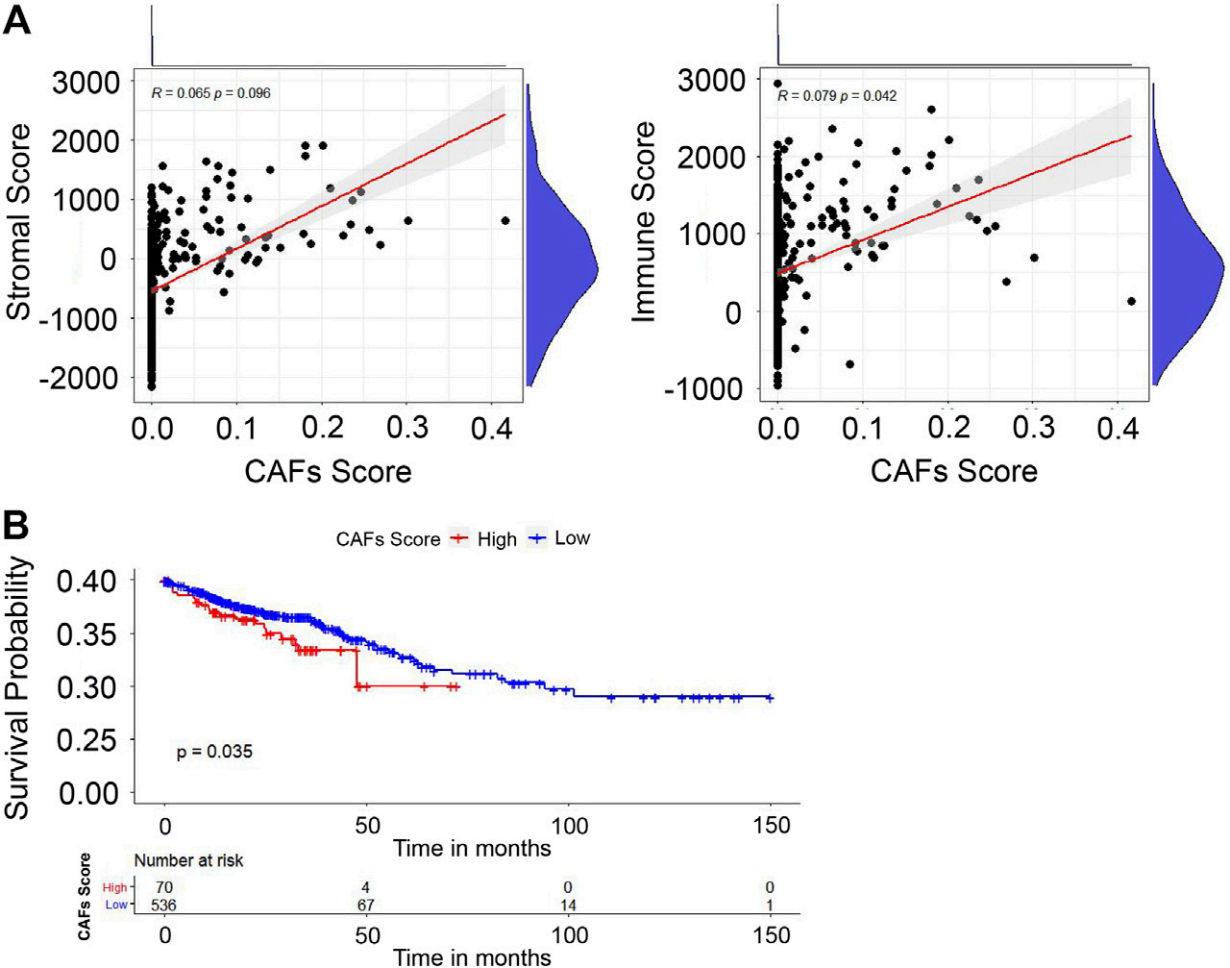
Evaluation of stromal/immune scores in CRC tissues. **(A)** The correlation between the stromal/immune scores and CAFs infiltration score. **(B)** The correlation between CAFs infiltration score and OS.

We first investigated the relationship between the stromal/immune scores and CAFs infiltration score. The results in Figure 3A reveal that stromal score was not correlated with CAFs infiltration score; however, the immune score showed a positive correlation with the CAFs infiltration score (*p* = 0.042). To investigate the potential relationship between OS and CAFs infiltration score, we further divided CRC patients into high- and low-score groups based on infiltration scores and the constructed Kaplan-Meier survival curves. We found that CAFs infiltration score was significantly negatively correlated with OS (*p* = 0.035) (Figure 3B). Overall, all these results suggested that CAFs infiltration score is associated with CRC patients’ prognoses.

### WGCNA for key gene module associated with CAFs

**FIGURE 4:**
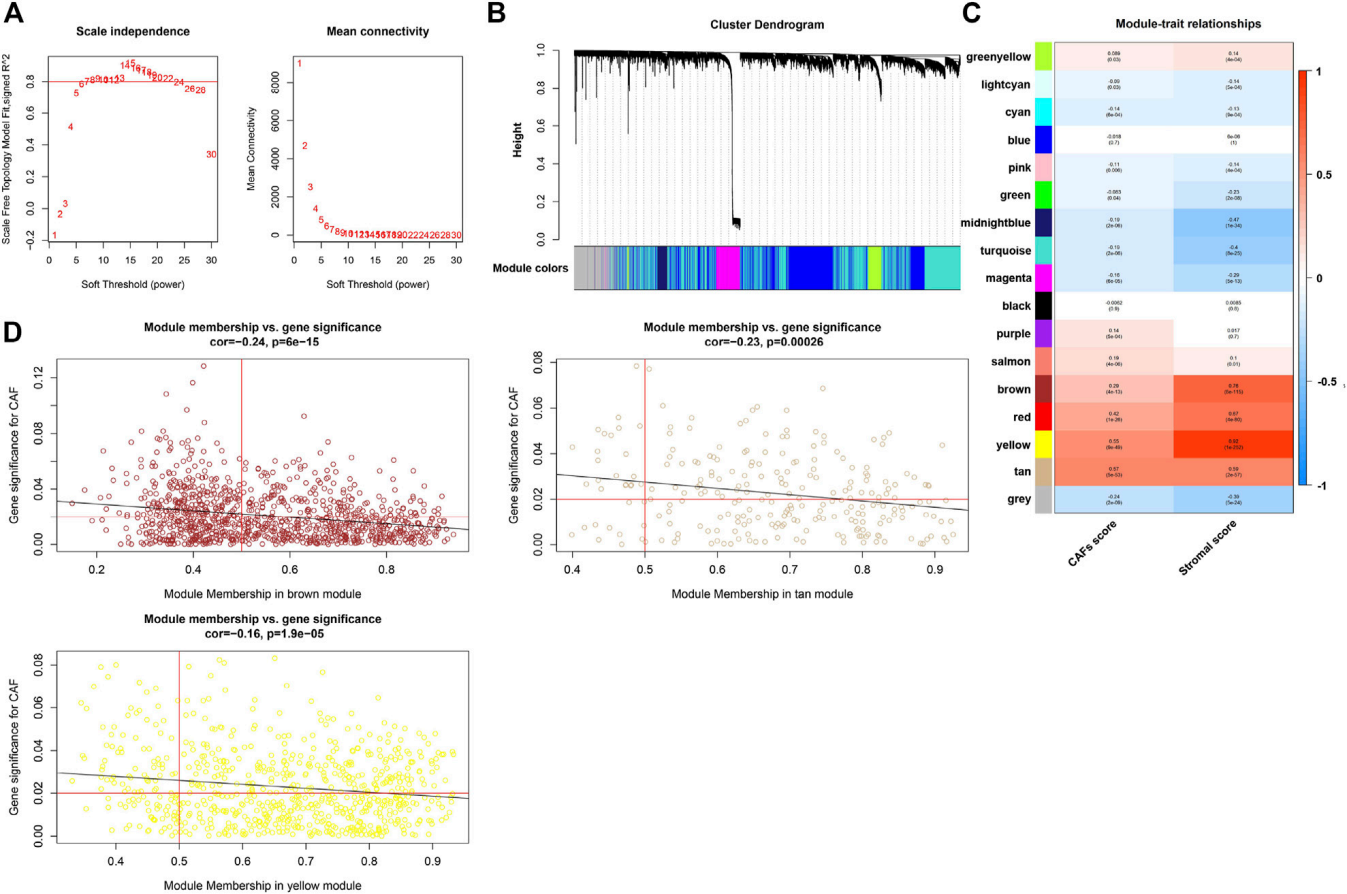
WGCNA and the identification of CAFs-related hub module. **(A)** Analysis of the scale-free ft index for various soft-threshold powers (β) and the mean connectivity for various soft-threshold powers. **(B)** Clustering dendrogram of all genes with dissimilarity based on topological overlap and assigned module colors. **(C)** The correlation between modules and traits were displayed. **(D)** The correlation between GS and MM in the brown, yellow, and tan modules.

We constructed the WGCNA analysis for all genes. With the power value selected as 7, the scale independence approached 0.8 (Figure 4A), suggesting a gene coexpression network with an inherent modularity and a scale-free topology. A total of 17 modules were identified through hierarchical clustering (Figure 4B). Next, we examined the correlation between the 17 modules and CAFs/stromal scores (Figure 4C). All of the brown, yellow, and tan modules had higher correlation with the CAFs/stromal scores. Thus, they were considered as key modules because of the high correlation with traits. Under the condition of module membership (MM) > 0.5 and gene significance (GS) > 0.03, 559 genes in the brown, yellow and tan modules were taken out (Figure 4D).

### GO and KEGG functional downstream analyses of CAFs-related genes

**FIGURE 5:**
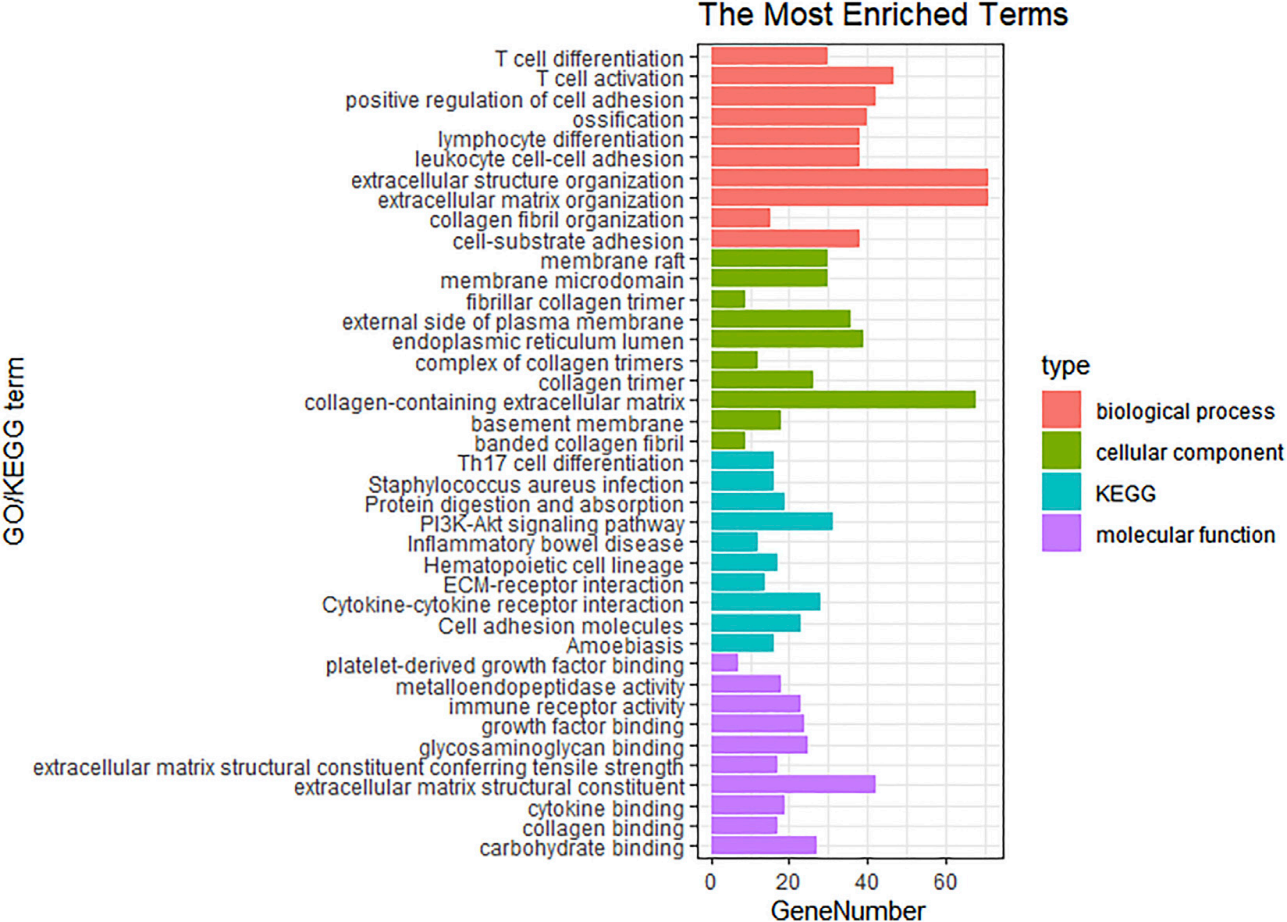
GO and KEGG analysis for the genes of brown, yellow and tan modules.

In order to investigate the biological functions and pathways of the above 559 genes in key modules, GO and KEGG pathway enrichment analyses were carried out. As shown in Figure 5, extracellular matrix organization, collagen fibril organization, collagen-containing extracellular matrix, and extracellular matrix structural constituent were the main significantly enriched GO terms. Moreover, the top 10 enriched KEGG pathways were also exhibited, which included Th17 cell differentiation, *Staphylococcus aureus* infection, protein digestion and absorption, PI3K-Akt signaling pathway, inflammatory bowel disease, hematopoietic cell lineage, ECM-receptor interaction, cytokine-cytokine receptor interaction, cell adhesion molecules, and amoebiasis.

### Alterations of CAFs-related genes in CRC samples

In order to investigate alterations in CAFs-related genes in the CRC samples, especially SNV and CNV, we analyzed CRC patients with SNV and CNV data after extraction from the TCGA. The top 30 genes with the highest mutation counts were shown in Supplementary Figure S1.

### Construction and verification of twenty-gene prognostic CAFs signature

**FIGURE 6:**
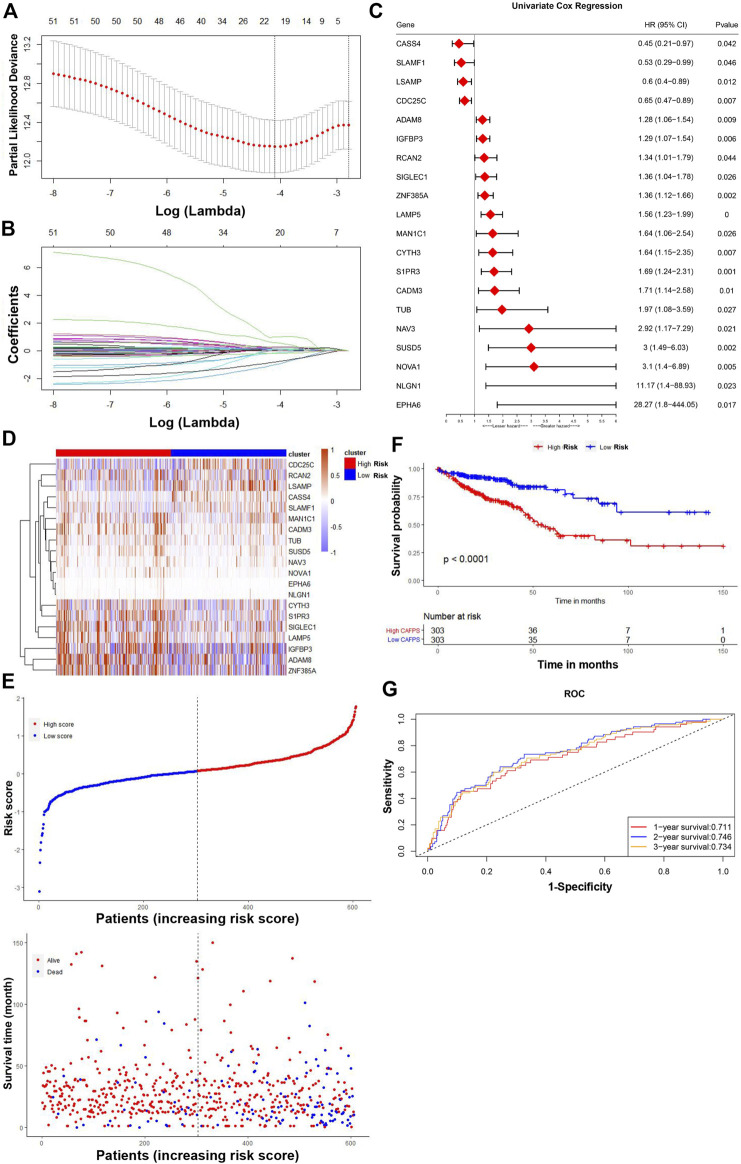
Screening of signature genes and the prognostic value of the CAFs-related signature in TCGA cohorts. **(A)** Ten-fold cross-validations for the screening of optimal parameter (lambda). **(B)** LASSO coefficient profiles determined by the optimal lambda. **(C)** The forest plot presented the HRs and *p*-values from the univariate Cox regression as well as the LASSO coefficient of the twenty prognostic signature genes. **(D)** Heatmap visualizing the expression levels of twenty prognostic CAFs genes with the CAFs risk scores in the TCGA cohort. **(E)** The distribution of patient survival status ranked by corresponding risk scores. **(F)** The Kaplan-Meier survival curves of OS between high and low risk score groups. **(G)** The time-dependent ROC curves of the prognostic signature for 1-, 2-, and 3-year overall survival.

In the TCGA cohort, by performing univariate Cox regression analysis on the 559 CAFs marker genes identified above, a total of 51 genes were found with *p* < 0.05. LASSO Cox regression algorithm was then performed to select hub genes. The minimal log(lambda) was determined as the optimal value by tenfold cross-validations (Figures 6A,B). Finally, a twenty-gene CAFs prognostic signature was constructed based on the expression levels of each gene and the coefficient, with the following formula: risk score = (0.0179849395327801 * expression of CYTH3) + (0.133678301953266 * expression of NAV3) + (0.97605105976877 * expression of EPHA6) - (1.01688005937021 * expression of CASS4) + (0.15388612377183 * expression of SIGLEC1)—(0.162312764949028 * expression of SLAMF1) + (0.0151723782273217 * expression of MAN1C1) + (0.107689308053911 * expression of LAMP5) + (0.0193478751759346 * expression of NOVA1) + (0.127100030231241 * expression of IGFBP3) + (0.0420090450866688 * expression of ADAM8)—(0.194826817771919 * expression of CDC25C) + (0.0834898746044337 * expression of ZNF385A) + (0.0492899593436562 * expression of CADM3) + (0.131501239268304 * expression of TUB) + (0.584400505367543 * expression of NLGN1) + (0.05334380595326 * expression of RCAN2) + (0.231247598432235 * expression of SUSD5) - (0.82291587246458 * expression of LSAMP) + (0.286394869616561 * expression of S1PR3). Among the 20 prognostic genes, sixteen (CYTH3, NAV3, EPHA6, SIGLEC1, MAN1C1, LAMP5, NOVA1, IGFBP3, ADAM8, ZNF385A, CADM3, TUB, NLGN1, RCAN2, SUSD5 and S1PR3) were regarded as risk-related genes, while CASS4, SLAMF1, CDC25C and LSAMP were considered as protective genes (Figure 6C). Kaplan-Meier survival curves revealed the relationship between prognosis and the expression levels of 20 genes (Supplementary Figure S2). Based on this risk formula, we calculated the CAFs risk score for each patient. The heatmap exhibited the risk scores and expression differences between the 20 genes in the TCGA cohort (Figure 6D).

**FIGURE 7:**
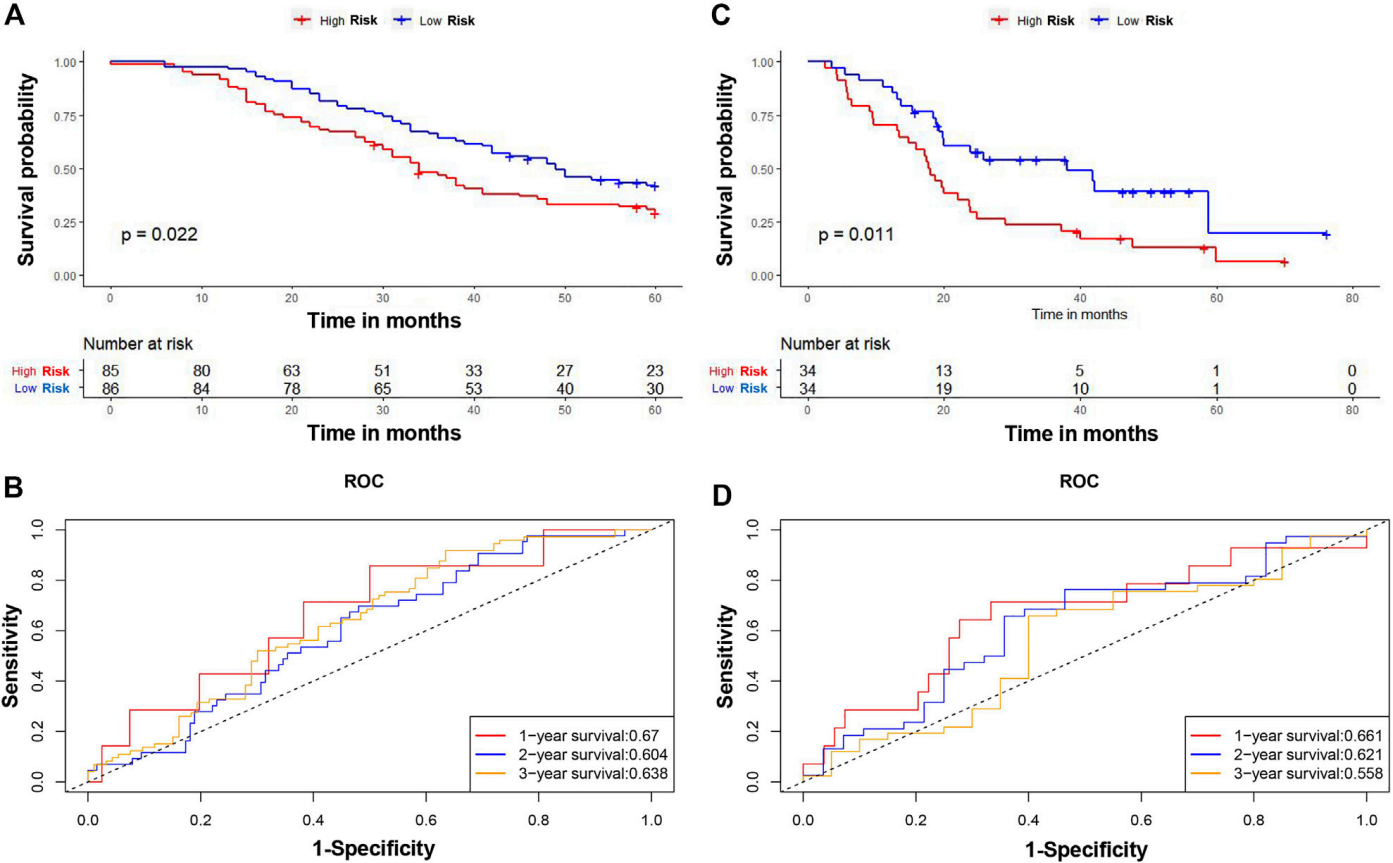
Verification of the CAFs-related signature. The Kaplan-Meier survival curves of OS between high and low risk score groups in GSE159216 **(A)** and GSE72968 **(C)**. The time-dependent ROC curves of the prognostic signature for 1-, 2-, and 3-year overall survival in GSE159216 **(B)** and GSE72968 **(D)**.

The patients in the TCGA cohort were divided into high- and low-CAFs risk groups according to the median risk scores. The distribution of the risk score and patients’ survival status were ranked by the risk score value (Figure 6E). According to the Kaplan-Meier survival curves, patients in the high CAFs risk group had significantly unfavorable OS compared with the low CAFs risk group (Figure 6F). A ROC curve was constructed in Figure 6G showing the prognostic accuracy of the signature, and the AUCs for 1-, 2-, and 3-year overall survival were 0.711, 0.746, and 0.734, respectively. Moreover, we also verified the predictive ability of the signature in another two independent cohorts, GSE159216 and GSE72968. Patients in the high CAFs risk group had a worse prognosis than those in the low CAFs risk group (Figures 7A–D).

### Independent prognostic value of CAFs risk score

**FIGURE 8:**
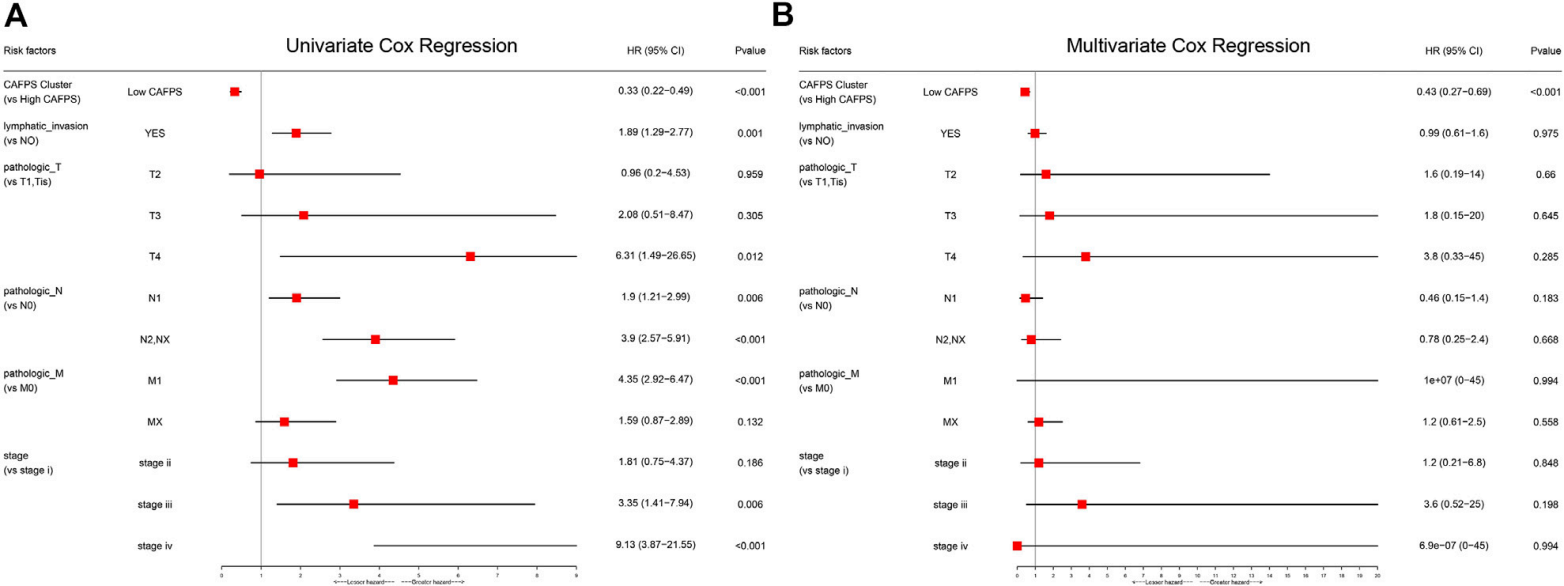
Univariate **(A)** and multivariate **(B)** Cox analysis and forest plot revealed the independent prognostic value of CAFs prognostic signature (CAFPS).

There were no differences in CAFs risk score between COAD and READ patients from the TCGA cohort (Supplementary Figure S3A). However, the CAFs risk score was significantly related to clinical stage, TNM stage, and lymphatic invasion (Supplementary Figures S3B–F). We next performed univariate and multivariate Cox regression analyses on the clinical variables to identify whether CAFs prognostic signature (CAFPS) was an independent prognostic predictor of OS. We found that CAFPS was significantly associated with OS in the univariate Cox regression analysis (HR = 0.33; 95% CI = 0.22–0.49; *p* < 0.001; Figure 8A). Furthermore, multivariate Cox regression analysis was carried out to correct the confounding factors. The CAFPS was nevertheless proved to be an independent predictor for OS (HR = 0.43; 95% CI = 0.27–0.69; *p* < 0.001; Figure 8B).

### Establishment of predictive nomogram for CRC patients

**FIGURE 9:**
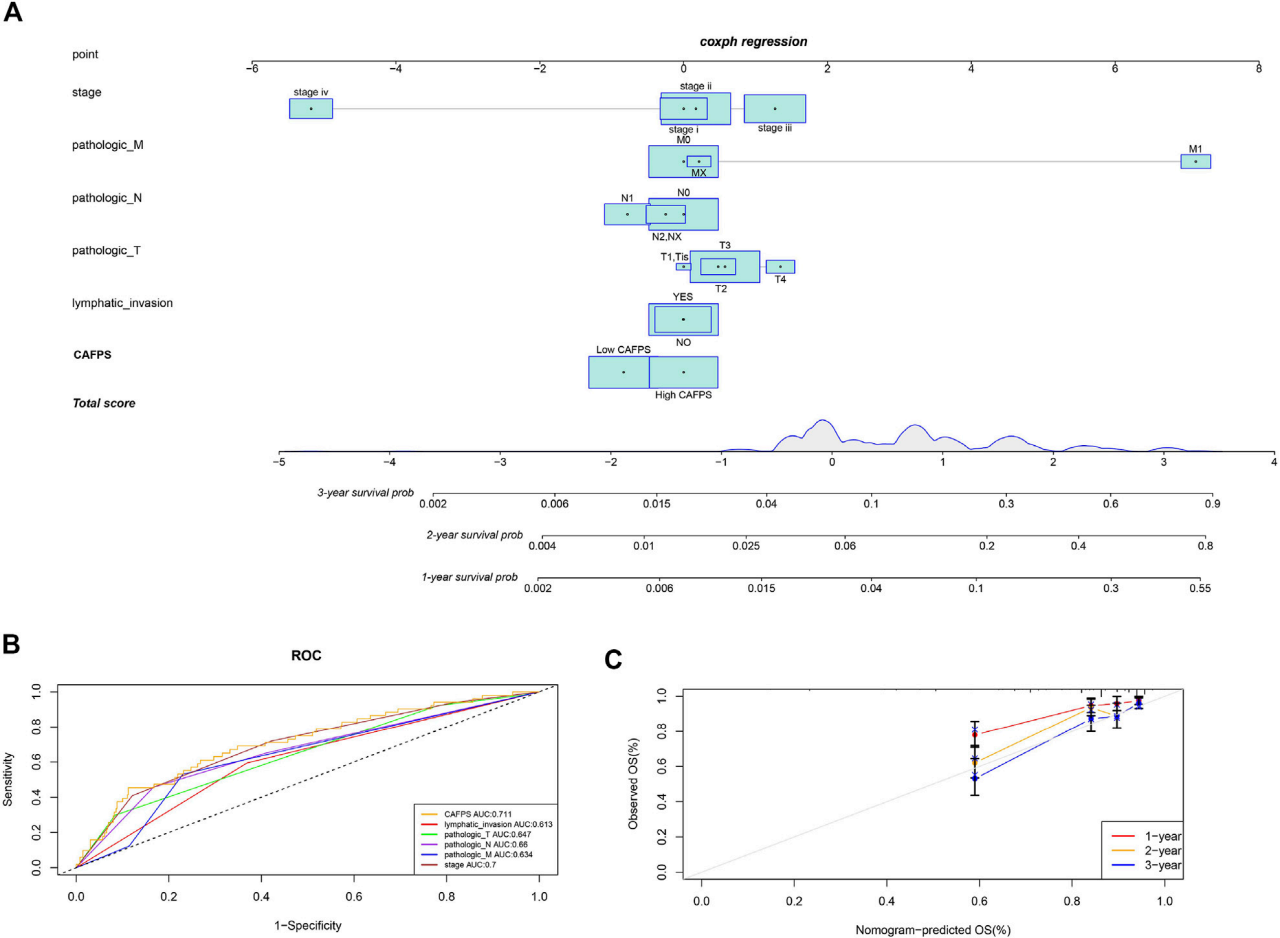
A nomogram was constructed to predict the survival of CRC patients in the TCGA cohort. **(A)** The nomogram for predicting the overall survival of CRC patients at 1, 2, and 3 years. **(B)** The ROC curves of the nomogram, clinical stage, TNM stage, and lymphatic invasion for the survival prediction of CRC patients at 1, 2, and 3 years. **(C)** The nomogram calibration curves of 1-, 2-, and 3-year survival probabilities.

According to the regression analysis results, we developed a nomogram including our CAFPS and multiple clinical factors. In the TCGA cohort, clinical stage, TNM stage, lymphatic invasion, and CAFPS were eventually selected to establish an accurate predictive nomogram (Figure 9A). Next, we evaluated the discriminative ability of the nomogram using the ROC-related AUC. The AUC of CAFPS was 0.711, and the calibration plots of 1-, 2-, and 3-year OS showed no deviations from the Platt calibration curves, indicating the high predictive accuracy of the nomogram (Figures 9B,C).

### Prognostic value of CAFPS for drug response

**FIGURE 10:**
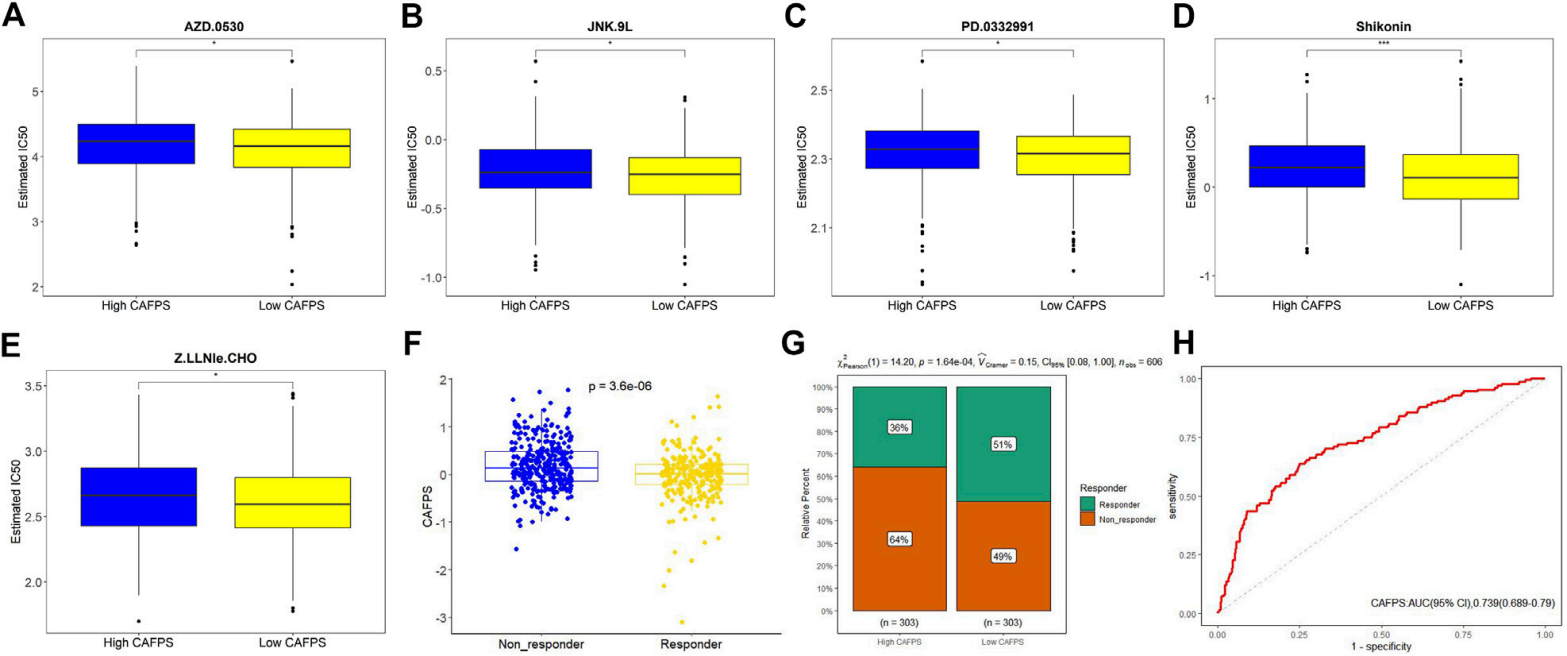
Drug sensitivity prediction in CRC patients. **(A–E)** Boxplot showing the mean differences in the estimated IC50 values of five drugs (AZD.0530, JNK.9L, PD.0332991, shikonin, and Z.LLNle.CHO). **(F)** Distribution of TIDE value after prediction. **(G)** Responders to immunotherapy in the low and high CAFPS groups. **(H)** ROC curve of CAFPS for 1-year overall survival.

We next examined the correlation between CAFPS and cancer hallmark-related pathways. As shown in Supplementary Figure S4, CAFPS was significantly associated with 32 cancer relevant pathways among the total of 50 pathways. To explore the difference between low-risk and high-risk groups regarding drug resistance potential, we estimated the IC50 levels of 138 chemotherapy drugs or inhibitors in the two groups. We found that AZD.0530, JNK.9L, PD.0332991, shikonin, and Z.LLNle.CHO could be candidate drugs for treating patients in the high-risk group (Figures 10A–E). The bubble chart shows the top 30 most relevant drugs for 20 prognostic genes (Supplementary Figure S5). We also predicted the response of CRC patients in the TCGA cohort to immunotherapy by the TIDE algorithm. The CAFPSs were significantly different between the non-responder group and the responder group (Figure 10F; *p* < 0.01). The proportion of responders in the low CAFPS group was significantly higher than that in the high CAFPS group (Figure 10G). The AUC of CAFPS for 1-year overall survival was 0.739 (95% CI = 0.689–0.79; Figure 10H). These evidences indicated that the CAFPS based on the signatures of 20 genes was helpful to assess patients’ response to chemotherapy and immunotherapy.

### Evaluation of the expression patterns of CAFs-related signature genes at the protein levels *via* HPA database

**FIGURE 11:**
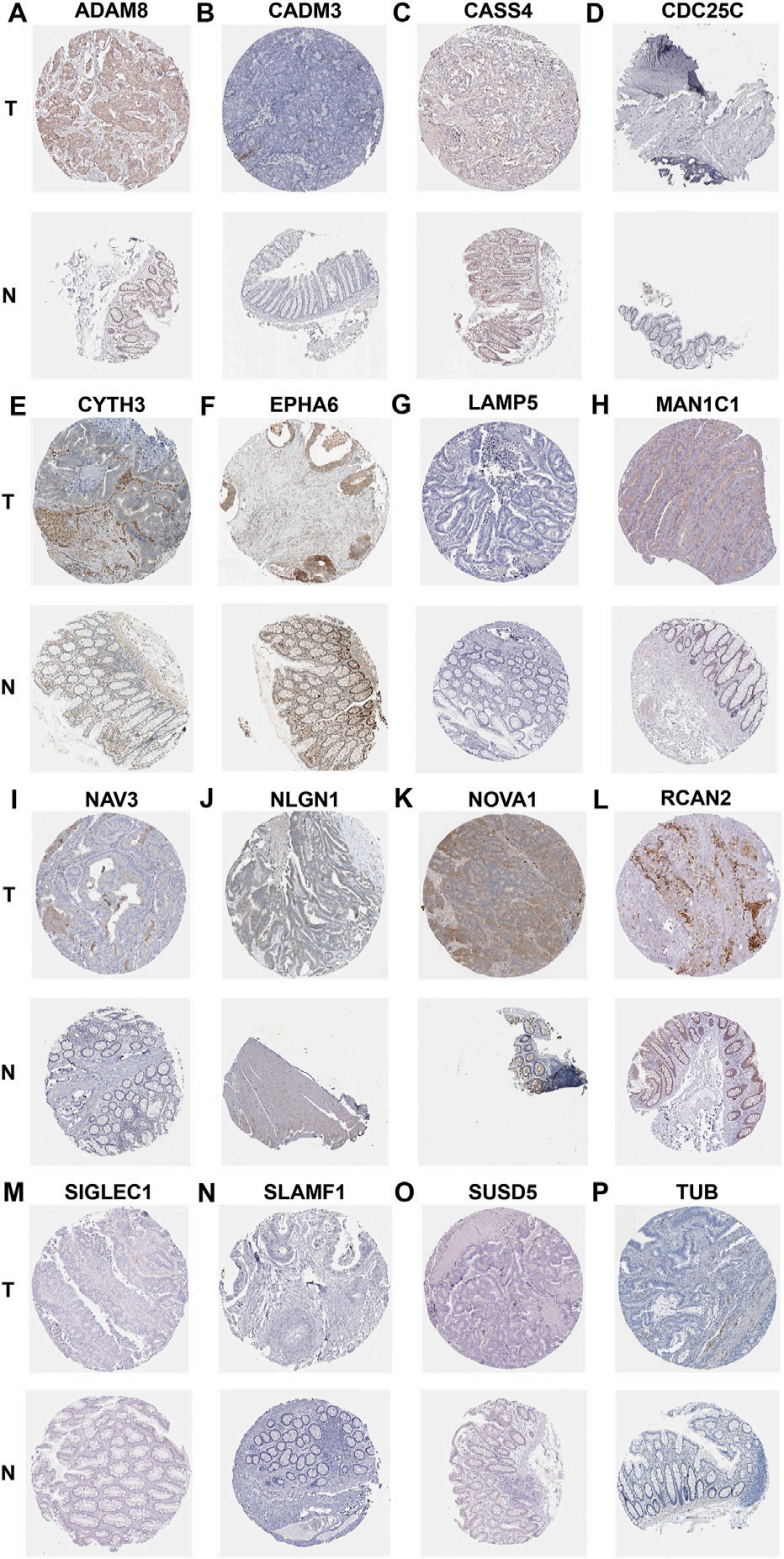
IHC showing the protein expression of ADAM8 **(A)**, CADM3 **(B)**, CASS4 **(C)**, CDC25C **(D)**, CYTH3 **(E)**, EPHA6 **(F)**, LAMP5 **(G)**, MAN1C1 **(H)**, NAV3 **(I)**, NLGN1 **(J)**, NOVA1 **(K)**, RCAN2 **(L)**, SIGLEC1 **(M)**, SLAMF1 **(N)**, SUSD5 **(O)**, and TUB **(P)** based on the HPA dtabase.

Finally, we used the IHC data from the HPA database to validate our previous findings to evaluate the expression of risk model genes associated with CAFs in tumor and normal tissues. Since IGFBP3, LSAMP, S1PR3 and ZNF385A have not been included in the HPA database, we provided the IHC results for ADAM8, CADM3, CASS4, CDC25C, CYTH3, EPHA6, LAMP5, MAN1C1, NAV3, NLGN1, NOVA1, RCAN2, SIGLEC1, SLAMF1, SUSD5, and TUB. The results showed that the protein expression levels confirmed the majority of our previous findings at the mRNA levels. Moreover, the IHC results from HPA database indicated that the protein expressions of ADAM8, CYTH3, and TUB were higher in CRC stroma (Figures 11A,E,P), while those of CADM3, EPHA6, MAN1C1, NAV3, NLGN1, NOVA1, and RCAN2 were higher in CRC interstitial areas (Figures 11B,F,H–L). No expressions of LAMP5, SLAMF1, and CDC25C were observed either in stroma or interstitial areas (Figures 11G,N,D). Moreover, CASS4, SIGLEC1, and SUSD5 were weakly expressed, like CASS4 and SUSD5 in interstitial areas (Figures 11C,O) and SIGLEC1 in stroma (Figure 11M).

## Discussion

CAFs, as one of the most abundant cell type in the TME, facilitate the development, propagation and invasiveness of tumors (Chan et al., 2017). They have been previously reported to support the TME, which can lead to poor prognosis and drug resistance (Chen et al., 2014; Shiga et al., 2015). On the one hand, CAFs can produce proteases to remodel the tumor extracellular matrix (ECM) and increase the stiffness of tumor tissue, creating a pathway for tumor cells to invade more easily (Kechagia et al., 2019). Increased tumor tissue stiffness in ECM also makes blood vessels collapse and lead to hypoxia, which promotes the survival and proliferation phenotype of tumor cells and reduces drug releases (Zhang et al., 2021). On the other hand, the roles of CAFs are not limited to ECM regulation but also include communicating with other cells to establish an immunosuppressive TME, enabling tumor cells to evade antitumor immunity (Barrett and Pure, 2020). CAFs are a rich source of secretomes and may influence the process of tumor-specific immune cell differentiation. Recently, several studies have indicated that CAFs skew infiltrating tumor-associated macrophage (TAM) populations towards the M2 immunosuppressive phenotype (Mao et al., 2021). However, emerging evidence suggests that WNT-2 secreted by CAFs may inhibit the differentiation and activation of dendritic cells, facilitating immune evasion by esophageal squamous cell carcinoma and colorectal cancer (Huang et al., 2022). Notably, the secretory function of CAFs could produce extensive crosstalk with surrounding cells in the TME, eventually leading to drug resistance (Monteran and Erez, 2019). In recent years, many studies have demonstrated the cancer-promoting effect of CAFs; therefore, the ability to target CAFs could be an attractive strategy for anti-cancer therapy.

In the present study, *via* the analysis of transcriptome data of TCGA cohorts, we estimated the proportion of CAFs in patients with CRC, and confirmed that it was closely associated with prognosis. Since CAFs exhibit a high degree of heterogeneity (Kalluri, 2016), integrating multiple biomarkers into an aggregated model would considerably improve their prognostic value. Thus, we established a CAFs-related gene signature including twenty genes (ADAM8, CADM3, CASS4, CDC25C, CYTH3, EPHA6, IGFBP3, LAMP5, LSAMP, MAN1C1, NAV3, NLGN1, NOVA1, RCAN2, S1PR3, SIGLEC1, SLAMF1, SUSD5, TUB, and ZNF385A) through WGCNA, univariate, LASSO, and multivariate Cox regression analysis for predicting the prognosis and therapy response of CRC patients. In our study, the risk score derived from CAFs-related gene signature was abbreviated as CAFPS in our study. Moreover, CAFPS’ predictive value has been validated in two additional independent cohorts, suggesting the reliability of the CAFPS-based model. We subsequently generated a nomogram based on clinical stage, TNM stage, lymphatic invasion, and CAFPS for clinical application.

Twenty CAFs-related genes were used to construct a new prognostic model through WGCNA. According to the risk value of each gene, ADAM8, IGFBP3, RCAN2, SIGLEC1, ZNF385A, LAMP5, MAN1C1, CYTH3, S1PR3, CADM3, TUB, NAV3, SUSD5, NOVA1, NLGN1, and EPHA6 were regarded as risk genes related to the poor prognosis of patients with CRC, whereas CASS4, SLAMF1, LSAMP, and CDC25C were associated with favorable prognosis. The biological functions of these genes involved in our signature have been elucidated more or less in previous studies. It was shown that the high levels of ZNF385A, LAMP, CADM3, NAV3, and NLGN1 indicate the poor prognosis of CRC patients (Martinez-Romero et al., 2018; Chang et al., 2021; Chen et al., 2021; Yu et al., 2021; Li et al., 2022), and these results were in accordance with our findings. Similarly, low expressions of CYTH3, NOVA1, and EPHA6 were highly correlated with longer OS in patients with other types of cancers (Zhang et al., 2014; Zhou et al., 2018; Xu et al., 2022). Furthermore, high expression of ADAM8 has been reported in various tumor types and is related to invasiveness and poor prognosis (Conrad et al., 2019). ADAM8 has been found to cleave and remodel the ECM components of the tumor stroma (Zack et al., 2009; Schlomann et al., 2015), and thus could directly contribute to tumor invasiveness and metastasis. IGFBP3 and SUSD5 promote epithelial-mesenchymal transition (EMT) through the upregulation of a major cell surface receptor of hyaluronic acid (CD44H) (Vicent et al., 2008; Du et al., 2022). SIGLEC1 is a sialic binding receptor mainly expressed by macrophages; the infiltration of SIGLEC1^+^ macrophages in CRC was associated with tumor progression (Cassetta et al., 2019). In addition, the involvement of S1PR3 has been demonstrated in tumor growth. The S1P/S1PR3 axis is considered to promote tumor cell proliferation, migration and angiogenesis (Lee et al., 2017).

In the present study, RCAN2 was identified as a harmful predictor; however, our results are the contrary to the finding of Niitsu et al. (Niitsu et al., 2016) This may be related to the KRAS mutation in CRC leading to the decreased expression of RCAN2. Similar to our results, low expressions of CASS4 and LSAMP indicated poor prognosis in other types of cancers (Zhao et al., 2020; Gong et al., 2022). SLAMF1 and CDC25C were also identified as anti-tumor biomarkers in CRC (Qi et al., 2021; Song et al., 2022).

Some limitations of our results have to be recognized. First, although some genes were expressed at very low levels in CRC tissues, 20 candidate hub genes could not be filtered out due to the restrictions of the applied bioinformatics methods. Second, ours was a retrospective study for the establishment of gene signatures using public databases, thus multi-center and large-sample studies are needed to prospectively verify the prognostic and predictive efficacy of our CAFPS. Finally, the verification by detection at the protein level is insufficient; the molecular mechanisms of how the 20 candidate genes of this study influence the prognosis of CRC patients and responses to treatments need to be further explored through basic research.

## Conclusion

In summary, we used WGCNA analysis to create a gene co-expression network, and identified and validated a twenty-gene CAFs-related signature associated with CRC progression and prognosis. Based on this signature, the CAFPS could identify CRC patients who might not benefit from chemotherapy or immunotherapy.

## Data Availability

The original contributions presented in the study are included in the article/Supplementary Material, further inquiries can be directed to the corresponding author.

## Supplementary material

Supplementary Figure S1 Alterations of CAFs-related genes in CRC patients from TCGA.

Supplementary Figure S2 The Kaplan-Meier survival curves of OS between highly expressed and lowly expressed CAFs-related genes.

Supplementary Figure S3 The differences in CAFs risk scores between different cancer types **(A)**, clinical stages **(B)**, lymphatic invasion **(C)**, and TNM stage **(D,F)**.

Supplementary Figure S4 Correlation between CAFPS and 50 cancer hallmark-related pathways.

Supplementary Figure S5 The top 30 most relevant drugs for 20 prognostic genes were shown by a bubble chart.
