# Supplementary figures and images for: Cancer-associated fibroblast-related gene signatures predict survival and drug response in patients with colorectal cancer
Source: Front Genet. 2022 Nov 25;13:1054152. doi: 10.3389/fgene.2022.1054152 (PMC9732269; doi:10.3389/fgene.2022.1054152)

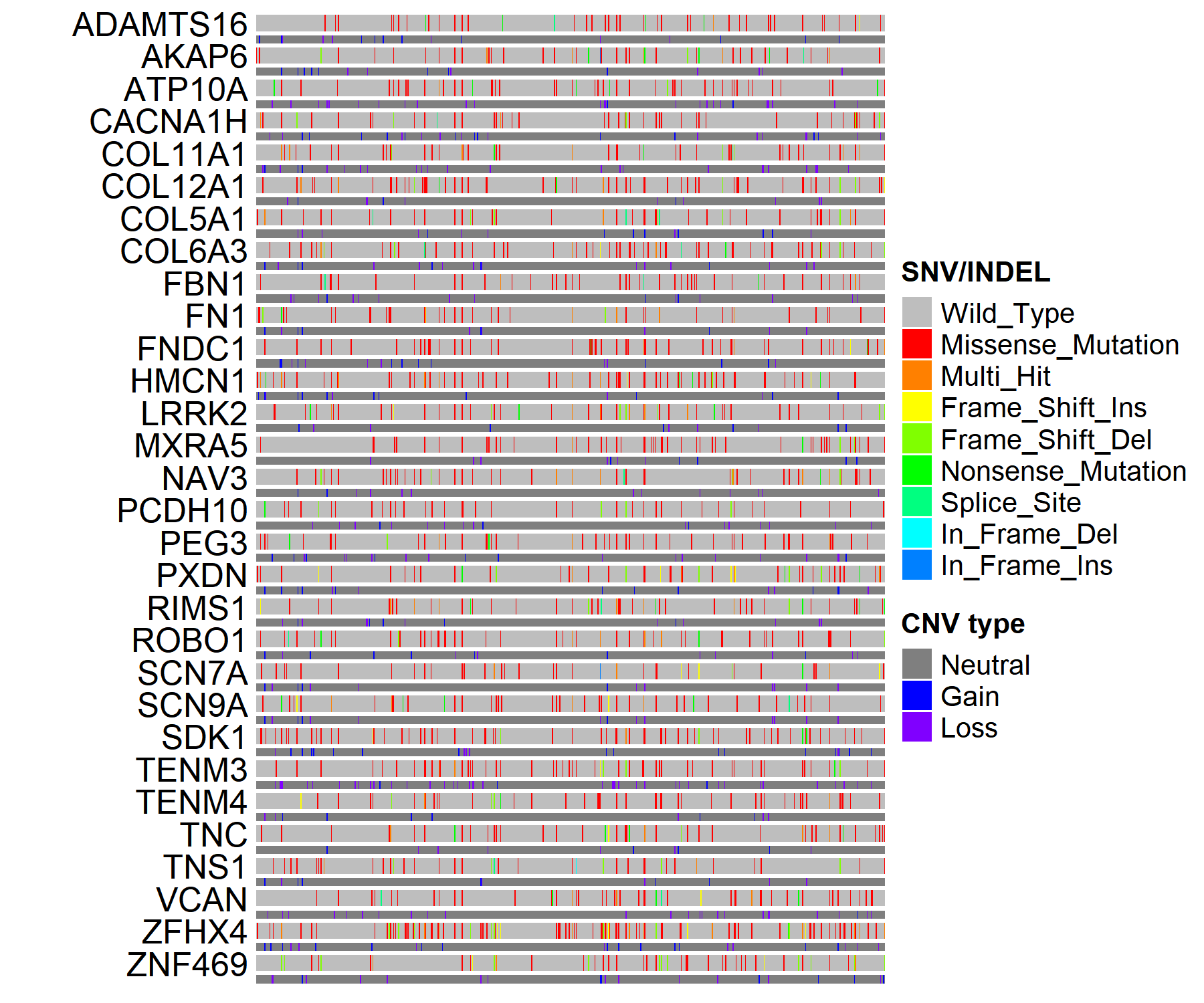

Supplement: Supplementary file 1 [file Image1.TIFF]

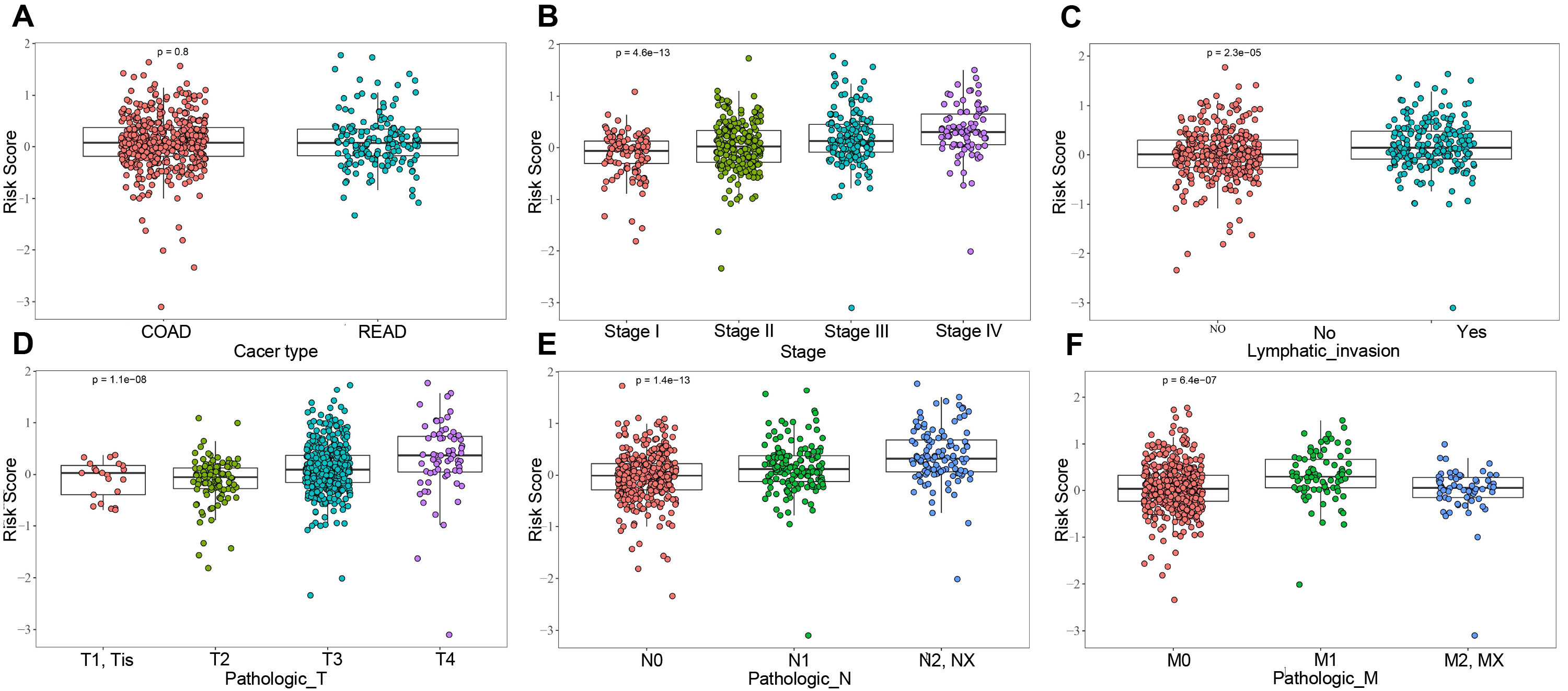

Supplement: Supplementary file 2 [file Image3.TIF]

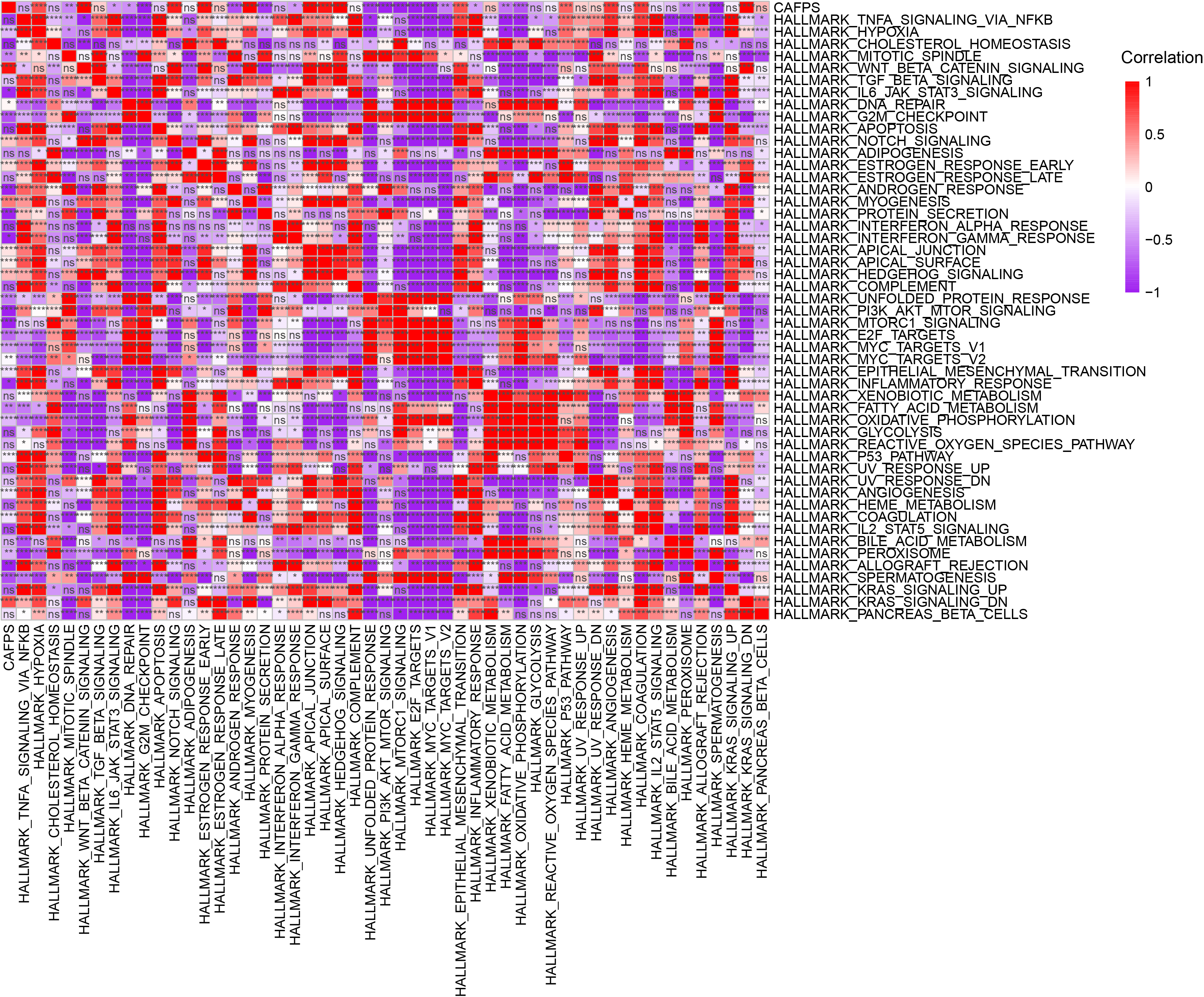

Supplement: Supplementary file 3 [file Image4.TIF]

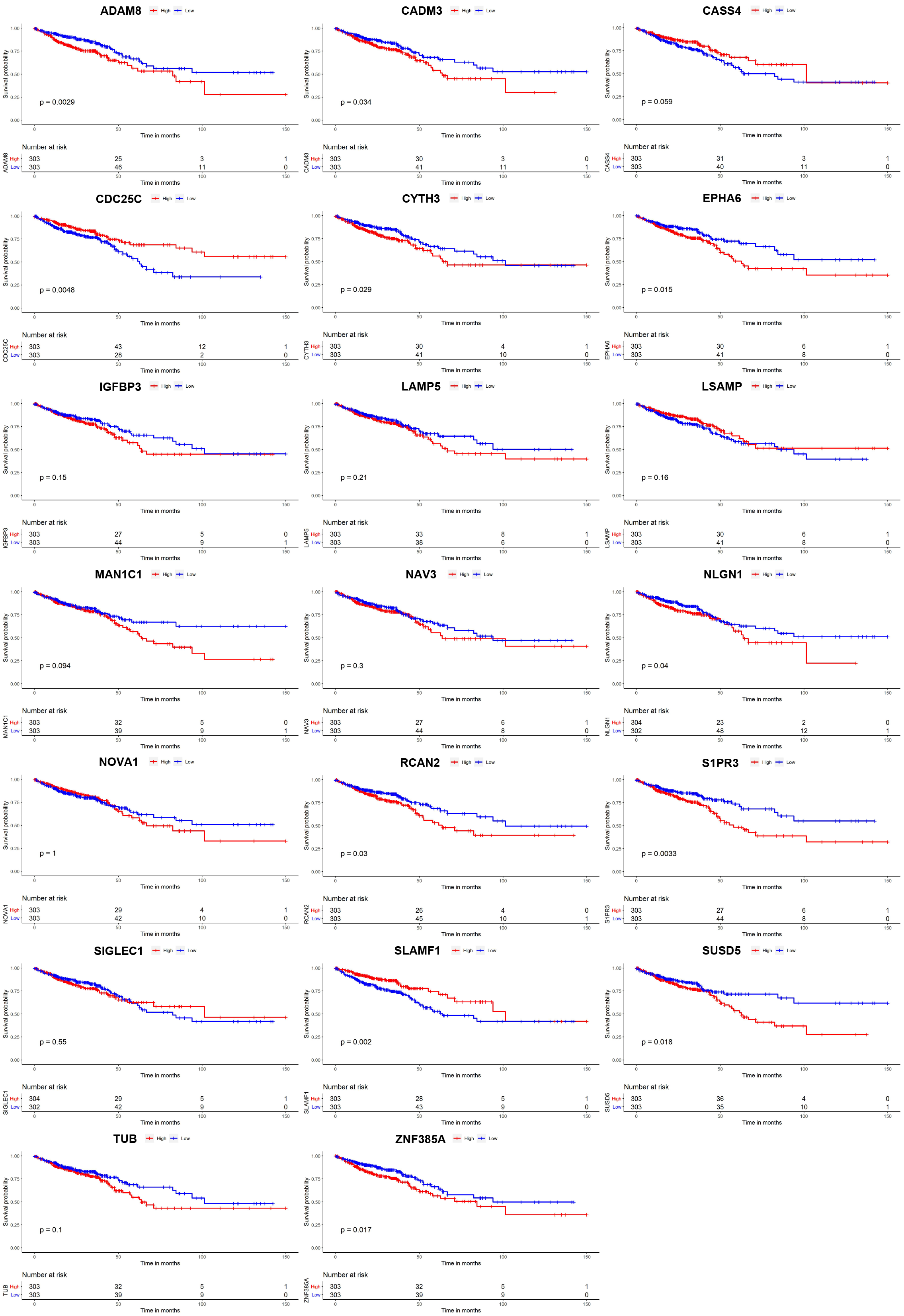

Supplement: Supplementary file 4 [file Image2.TIF]

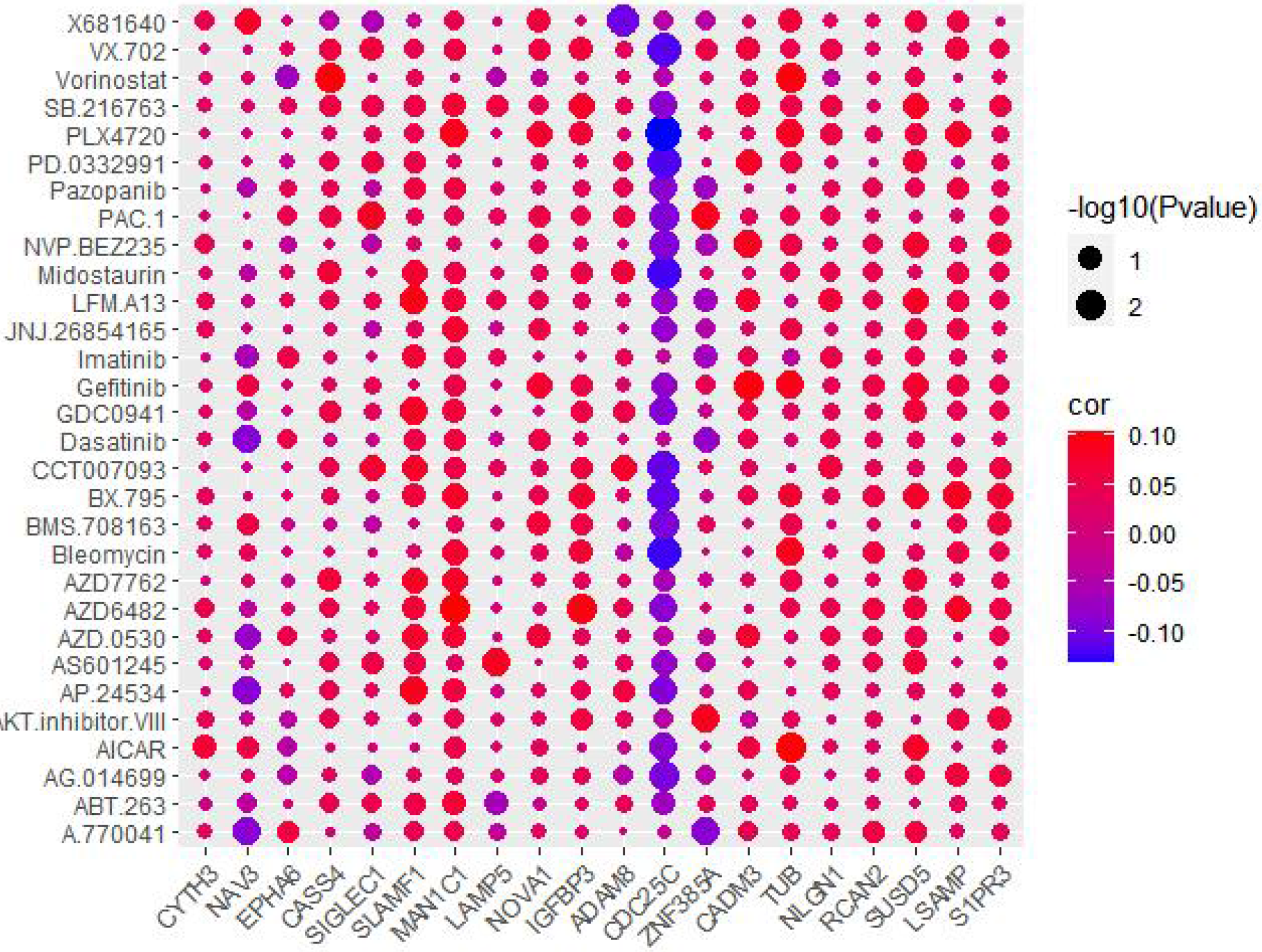

Supplement: Supplementary file 5 [file Image5.TIF]
